# 25-Hydroxyvitamin D, Vitamin D Binding Protein, Bioavailable 25-Hydroxyvitamin D, and Body Composition in a Diverse Sample of Women Collegiate Indoor Athletes

**DOI:** 10.3390/jfmk5020032

**Published:** 2020-05-09

**Authors:** Jennifer B. Fields, Sina Gallo, Jenna M. Worswick, Deanna R. Busteed, Margaret T. Jones

**Affiliations:** 1 Frank Pettrone Center for Sports Performance, George Mason University, Fairfax, VA 22030, USA; jfields8@gmu.edu (J.B.F.); sgallo2@gmu.edu (S.G.); dbusteed@gmu.edu (D.R.B.); 2 School of Kinesiology, George Mason University, Manassas, VA 20110, USA; worswick23@me.com; 3 Nutrition and Food Studies, George Mason University, Fairfax, VA 22030, USA

**Keywords:** bone mineral density, skin pigmentation, sun exposure, vitamin D, sport

## Abstract

Women athletes are at higher risk for bone diseases; yet, information on vitamin D status ((25(OH)D), vitamin D binding protein (VDBP), and bioavailable 25(OH)D is limited. Collegiate athletes (*n* = 36) from volleyball (WVB), basketball (WBB), and track and field (WTF) were measured for (25(OH)D), VDBP, and bioavailable 25(OH)D; body composition and bone mineral density (BMD); and skin pigmentation. Participants self-reported daily vitamin D intake and sun exposure. One-way analysis of variance analyzed mean differences in measures across sports. Linear regression examined relationships between 25(OH)D; VDBP; bioavailable 25(OH)D; and whole body, hip, and spine BMD. Participants’ (mean ± SD, 19.4 ± 1.4 years, 172.75 ± 8.21 cm, 70.9 ± 13.2 kg, and 22.9 ± 4.1% body fat) overall mean 25(OH)D was 70.5 ± 32.25 nmol/L, and 28% of participants were deemed inadequate and 61% below thresholds identified as sufficient for athletes. Although WBB athletes consumed higher (*p* = 0.007) dietary vitamin D (760.9 ± 484.2 IU/d) than WVB (342.6 ± 257.8) and WTF (402.3 ± 376.4) athletes did, there were no differences across sport in serum 25(OH)D. WVB and WTF had higher bioavailable 25(OH)D than WBB. No relationships existed between vitamin D status and body composition. Vitamin D inadequacy was identified among 1/3 of women indoor sport athletes. Consistent monitoring of vitamin D status and diet are recommended to sustain athlete health and sport performance.

## 1. Introduction

Vitamin D is an essential fat-soluble vitamin that plays a well-recognized role in bone heath, immune function, neuromuscular function, mood state, and overall physical performance [1,2]. It is directly related to muscle strength, mass, and function, which are crucial factors in the reduction of injury and successful physical performance [1,2]. Cholecalciferol (vitamin D_3_) is synthesized in the skin via ultraviolet B-light exposure, and is found in natural animal food sources and through fortified foods or supplementation [3]. Even though sun exposure may be a way to obtain adequate vitamin D, factors such as skin pigmentation, sunscreen, northern latitudes, and excess adiposity may reduce vitamin D synthesis and its bioavailability [3]. Ergocalciferol (vitamin D_2_) is a form of vitamin D found in vegetarian food sources and is commonly used as a dietary supplement. Once formed, vitamin D (regardless of form) is metabolized in the liver to 25-hydroxyvitamin D (25(OH)D), and is metabolized further by the kidney into its biologically active form, 1,25-dihydroxyvitamin D [3]; yet, 25(OH)D is the commonly accepted biomarker of vitamin D status.

Information on the vitamin D status of women collegiate athletes remains limited, despite being researched extensively within the general population. Findings from a previous study with collegiate athletes (*n* = 41: 18 men, 23 women) demonstrated that a vast majority of both indoor and outdoor sport athletes had insufficient vitamin D concentrations (defined as <75 nmol/L) during winter, thus heightening the risk of illness [4]. Weight-bearing exercises can help athletes build bone mineral density and lean body mass, which is important in the prevention of stress fractures and osteoporosis. However, women athletes who engage in sports that emphasize leanness and high levels of cardiovascular fitness are more likely to be affected by decreased bone mineral density, disordered eating, and menstrual cycle irregularities. Previous work among ethnically diverse male collegiate athletes found no relationship between bone mineral density (whole-body, spine, and hip) and serum 25(OH)D [2,5,6], indicating the possibility that these established relationships might not be applicable in athletes. Vitamin D binding protein (VDBP) is the primary vitamin D carrier, binding 85–90% of total circulating 25(OH)D, with the remaining unbound 25(OH)D considered to be free, or bioavailable [6]. Differentiating between 25(OH)D and bioavailable 25(OH)D is important. Those with darker skin may be at a higher risk for deficiency due to genetic differences in VDBP, resulting in greater concentration of bioavailable (free) 25(OH)D [2]. Consequently, it has been suggested that bioavailable 25(OH)D may have a better association with bone health among racially diverse athletes than serum 25(OH)D concentration [6,7]. 

Limited data exist in regard to the relationship between 25(OH)D, VDBP, and bioavailable 25(OH)D, among women collegiate athletes, who may be at higher risk for low bone mineral density. Considering the role of vitamin D in muscle mass and injury prevention, there is a need to assess such factors among this population. Therefore, the purpose of the current study was to (1) assess circulating 25(OH)D, VDBP, and bioavailable 25(OH)D status among women collegiate athletes; (2) examine differences in 25(OH)D, VDBP, bioavailable 25(OH)D, lean body mass, and bone mineral density, across women indoor sport athletes; and (3) determine the relationship of 25(OH)D, VDBP, and bioavailable 25(OH)D with lean body mass and bone mineral density. It was hypothesized that the women athletes would have lower vitamin D concentrations, and differences across sports would be apparent. Further, it was hypothesized that higher levels of 25(OH)D and bioavailable 25(OH)D would be positively related to lean body mass and bone mineral density.

## 2. Materials and Methods 

This was a cross-sectional study among National Collegiate Athletic Association-Division I women indoor-sport athletes representing the sports of basketball, track and field, and volleyball. Participants were evaluated at one time point during the winter months (January, February). 

### 2.1. Participants

The participants (*n* = 36; Mean ± SD: 19.4 ± 1.4 years) were trained women collegiate athletes from three indoor sports: track and field (TF; *n* = 12), basketball (BB; *n* = 12), and volleyball (VB; *n* = 12). Participants followed sport-specific training regimens that required participation in regular sport-specific training activities with specific neuromuscular demands. All were cleared for intercollegiate athletic participation by the university sports medicine staff prior to completing a medical history form. Following an explanation of the risks and benefits associated with participation, a written consent form was signed by each participant. Procedures were approved by the George Mason University Institutional Review Board for Human Subjects (Protocol 978815-5, 20 December 2016). Consuming > 400 IU/d of vitamin D supplementation [8] or pregnancy were grounds for exclusion. 

### 2.2. Measurements 

Self-report questionnaires, in regard to demographics, travel within the previous three months, sport experience, and sunscreen use were completed by participants before data collection was initiated. Ethnicity was self-reported by participants.

### 2.3. Anthropometrics

The Tanita (SC-331S Total Body Composition Analyzer, Tanita corporation of America, Inc. Arlington Heights, IL, USA) was used to measure body mass to the nearest 0.01 kg. A wall-mounted stadiometer (Detecto, Webb City, MO, USA) measured body height to the nearest 0.1 cm. Body mass index ((BMI; weight (kg)/height (m^2^)) was calculated. Results were categorized as follows: underweight (BMI ≤ 18.5 kg/m^2^), normal weight (BMI 18.5–24.9 kg/m^2^), overweight (BMI 25–29.9 kg/m^2^), and obese (BMI ≥ 30 kg/m^2^).

### 2.4. Body Composition and Bone Health 

Dual energy X-ray absorptiometry (DXA, Hologic, Horizon A model, Hologic Inc., Waltham, MA)) was used to measure whole-body bone mineral density (BMD), body fat (BF%), fat mass (FM), and lean body mass (LBM). Z-scores were based on Hologic population-specific reference data [9]. An International Society for Clinical Densitometry (ISCD) Certified Bone Densitometry Technologist (CBDT) supervised all scans. During the test participants wore standard issue athletic gear (i.e., no metal), remained motionless, and breathed normally [10]. The radiation limits for X-ray exposure (~3.4 mSV) were not exceeded. For proper calibration of bone measures, a quality assurance phantom test (Hologic phantom serial #26436) was performed prior to each test [10]. Weekly calibration was performed using a whole-body phantom (Hologic #1104). Coefficients of variation were established in our laboratory as follows: lean body mass, 1.07%; body fat%, 1.17%; fat mass, 1.88%; and bone mineral density, 1.03%.

### 2.5. Vitamin D Intake

A Food Frequency Questionnaire (FFQ) specific to vitamin D intake was collected to assess dietary consumption and supplementation [11]. The FFQ totaled 20 items, including dairy products, protein sources, vitamin D fortified foods, and supplementation. The Registered Dietitian, with a Board Certification in Sports Dietetics, employed by the Intercollegiate Athletics program at the athletes’ university modified the FFQ to include commonly consumed food from the target population. The USDA Food Composition Database was used to calculate daily vitamin D (IU/day) via both diet and supplementation [12].

### 2.6. Skin Pigmentation

A portable computerized spectrophotometer (CM-600D, Konica Minolta) was used to measure skin pigmentation on the forehead [13]. The individual typological angle (ITA) {ITA° = [arc tangent (L* − 50)/b*] 180/3.14159} was calculated based upon the Commisssion Internationale de l’Eclairage colorimetry system (L*a*b*) [13]. Classification consisted of five skin phototypes: dark (≤10°), olive (10–28°), medium (28–41°), fair (41–55°), and very fair (>55°) [12].

### 2.7. Blood Sampling and Analyses

Blood samples were collected upon arrival to the laboratory. With participants seated in an upright position, samples were collected from an antecubital vein using standard sterile phlebotomy procedures. Blood was drawn into one 5-mL vacutainer tube that contained no additive (BD Biosciences, San Jose, CA, USA). Samples coagulated in cooling beds for approximately 20 min, then were centrifuged for 15 min at 2500 rpm (Eppendorf 5702R, Eppendorf North America, Hauppauge, NY, USA) before being stored at −80 °C until analysis. A second 5-mL vacutainer tube containing the anticoagulant ethylenediaminetetraacetic acid (EDTA) was collected, centrifuged as previously described, and the resultant supernatant was stored at −80 °C. Measurements of the serum concentration of 25(OH)D were performed in duplicate using a commercially available ELISA kit (Monobind, Lake Forest, CA) and a plate reader (Epoch, BioTek, Winooski, VT, USA). The plasma concentration of VDBP was measured in duplicate using a commercially available bead-based assay kit (EMD Millipore; Billerica, MA, USA) and a CCD-based Luminex Magpix (Austin, TX) multiplex system. The serum concentration of albumin was measured using a colorimetric end point assay (Pointe Scientific Inc. Canton, MI, USA and a ChemWell auto-analyzer (Awareness Technology, Palm City, FL, USA). Intra-assay coefficients of variation for 25(OH)D, VDBP, and albumin were 3.7%, 5.7%, and 3.0%, respectively. Bioavailable 25(OH)D was calculated according to previously published methods [2,6,7]. The following cutoffs were used to determine 25(OH)D (nmol/L) health status: ≤50 = inadequate, 50–75 = adequate for the healthy general public [14], and ≥75 = sufficient for athletic populations as suggested by others [15,16].

### 2.8. Statistical Analysis

SPSS Version 24.0 (IBM, Armonk, NY) was used for data analysis. The Shapiro–Wilks test was used to confirm normality of all variables. A natural log-transformation was applied prior to analysis for any non-normally distributed variable. Descriptive statistics are presented as Mean ± SD, and categorical variables as *n* (%). A one-way analysis of variance was used to assess mean differences in participant characteristics across sports teams. Multiple linear regression analyses, including sport team and skin pigmentation as covariates, were performed with body composition variables as the dependent variables. Parameter estimates along with 95% CI were reported. *p*-value was set at *p* < 0.05.

## 3. Results

Table 1 includes participant characteristics by sports team. Height, body mass, body mass index (BMI), body fat (BF)%, and fat mass (FM) were significantly different across teams. WTF showed the lowest height (*p* = 0.005), mass (*p* < 0.001), BMI (21.3 ± 1.4 kg/m^2^, *p* = 0.001), BF% (*p* = 0.001), and FM (*p* = 0.001), compared to both WVB and WBB. Whole-body, hip, and spine bone mineral density (BMD) did not differ across teams, with the exception of hip Z-score (*p* = 0.044).

Overall, 39% of athletes were vitamin D sufficient (99.28 ± 33.25 nmol/L), with 28% classified as inadequate (39.58 ± 6.13 nmol/L). No differences in 25(OH)D concentrations or VDBP were found across teams; however, WVB had the highest bioavailable 25(OH)D and WBB had the lowest (*p* = 0.012). There were no significant differences in skin pigmentation and sun exposure across teams, and no association existed between these variables and 25(OH)D levels (Table 1). 

Regression models revealed no statistically significant associations between 25(OH)D; VDBP; bioavailable 25(OH)D; and lean body mass, whole-body, hip, and spine BMD (Table 2).

## 4. Discussion

Vitamin D inadequacy was identified among ~1/3 women collegiate athletes and 2/3 below thresholds identified as sufficient for athletes. This observation is in support of previous findings with women athletes where rates of insufficiency (<75 nmol/L) were reported as 83% among gymnasts [17], 40% among endurance trained athletes [18], and 58% among elite track and field athletes [19]. Further, Japanese women indoor sport athletes from basketball and volleyball (*n* = 15; age: 20.6 ± 0.6 years; height: 162.6 ± 3.3 cm; weight: 60.0 ± 4.3 kg; and body fat %: 25.2 ± 2.7%) reported vitamin D values of 47.5 ± 10.0 nmol/L in samples collected during the winter [20]. These values are lower than those reported in the current study (70.5 ± 32.25 nmol/L), which may be a function of differences in body type of the Japanese athletes (i.e., shorter, lower body weight, and higher BF%). While dietary intake of Vitamin D was not assessed, few foods in Japan are vitamin D-fortified and the intake of fish has declined [21]. Further, sun exposure data were not collected. The length of solar exposure required to synthesize adequate vitamin D is >1 h in the winter months (November-March) in Japan, and it was unlikely the athletes achieved that amount [22]. Results from the current study showed minimal differences across sports teams, as well as no relationship between body composition measures (i.e., bone health and lean body mass) and vitamin D metabolites (i.e., 25(OH)D, VDBP, and bioavailable 25(OH)D).

While it is apparent that athletes, in particular women athletes, experience low 25(OH)D levels [17,18,19], less is known in regard to VDBP and bioavailable 25(OH)D status in this population. In healthy male (*n* = 27) and female (*n* = 22) college students (23.5  ±  3.4 years), average VDBP and bioavailable 25(OH)D were 54.66  ±  26.32 nmol/L and 9.58  ±  6.74 nmol/L, respectively [23]. In a large, diverse sample of male international athletes, Allison et al. [6] reported VDBP ranged from 370.5 to 478.5 μg/mL, and bioavailable 25(OH)D ranged from 0.7 ng/mL (severely deficient) to 4.7 ng/mL (sufficient), with athletes classified as “sufficient” having higher bioavailability levels [6]. In the women collegiate athletes from the current study, VDBP values ranged from 379 μg/mL to 495 μg/mL and bioavailable 25(OH)D ranged from 1.64 nmol/L to 9.67 nmol/L. The VDBP and bioavailable 25(OH)D were positively associated with 25(OH)D status, thus higher values were more prevalent in 25(OH)D sufficient athletes (>75 mol/L). Slight differences in reported values between the current study and those previously published studies may be a result of the different populations sampled, as Powe et al. did not assess athletes [23] and Allison et al. examined a large sample of male athletes across race (Arab (*n* = 327), Asian (*n* = 48), Black (*n* = 108), Caucasian (*n* = 53), and Hispanic (*n* = 68)) [6].

No association was found between 25(OH)D and whole-body BMD, hip BMD, and spine BMD. The relationship between 25(OH)D and BMD has been shown consistently in the general population [24], but this relationship may be sport-dependent for athletes. Due to the intense demands associated with optimal sport performance, athletes engage in excess amounts of weight bearing exercise training, and it is hypothesized that the high impact activity will increase BMD [25,26,27,28]. Training that invokes greater bone loading and multidirectional impact (i.e., volleyball, basketball, and track jumpers) may lead to enhanced osteogenic stimulus, thus increasing BMD [28,29]. It has been proposed that VDBP may help explain these inconsistencies reported in athletic populations [23,30,31]. In contrast to the limited studies on this topic, no predictability of whole body, hip, or spine BMD on 25(OH)D, VDBP, or bioavailable 25(OH)D was observed in the current study. However, the limited number of studies in which VDBP and bioavailable 25(OH)D were analyzed either had a large sample size (i.e., 604 athletes [6]) or assessed healthy general population adults [7,23].

Although sun exposure, skin pigmentation, and dietary vitamin D intake have been shown to be determinants of serum 25(OH)D [32,33], no relationships were observed in the current study. In part, this may be attributed to the time of year data were collected (winter months). Previous reports have also shown a lack of relationship between 25(OH)D despite abundant sun exposure [34,35,36], signifying that sun exposure may not be an adequate indicator of vitamin D status. Interestingly, skin pigmentation also had no association with 25(OH)D, despite prior studies in which those with darker skin tones had a greater likelihood of having a 25(OH)D deficiency [36,37]. It has been hypothesized that darker-skinned athletes are more inclined to vitamin D deficiencies due to large amounts of melanin in the epidermal layer [38]. Increased melanin reduces the capacity of the skin to synthesize vitamin D_3_, thus increasing risk of insufficiencies. 

Previous research has indicated that deficiencies do not always correlate with low consumption of dietary vitamin D (<400 IU/day) [39,40]. Athletes in the current study consumed from the diet, on average, 501.9 ± 417.4 IU/day. Interestingly, WBB consumed significantly higher vitamin D (760.9 ± 484.2 IU/day) than WVB (342.6 ± 257.8 IU/day) and WTF (402.3 ± 376.4 IU/day). The higher consumption in WBB athletes may be due to the routine consumption of a milk-based high protein recovery drink that contained 25% of the Recommended Dietary Allowance of vitamin D. Athletes consumed most of their vitamin D intake from dairy sources (i.e., milk and yogurt) and fish, and no athletes were supplementing at the time of the study. The amounts consumed by all athletes were likely too low to affect 25(OH)D status, and as such may help explain why no relationship between dietary intake and serum levels was observed [39].

Further, no relationship was observed between lean body mass and 25(OH)D, VDBP, or bioavailable 25(OH)D. Findings from previous research report significant associations between 25(OH)D and body mass (r = −0.57), %body fat (r = −0.54), fat mass (r = −0.60), and fat free mass (r = −0.51) (*p* < 0.05) in collegiate athletes (*n* = 42: 24 men, 18 women) [41,42]. It has been suggested that vitamin D is sequestered into excess adipose tissue, thereby leading to decreased bioavailability as a result of altered absorption and metabolism of vitamin D [43]. However, Forney et al. also saw no relationship between 25(OH)D and lean body mass and BF% in physically active college students (*n* = 40: 20 men, 20 women) [44]. Further, a negative association between 25(OH)D and body mass index has been reported across a variety of populations [44,45,46]. Research that examines the relationship between 25(OH)D and lean body mass in athletes from high-impact sport is limited and inconclusive.

The relationship of vitamin D status (25(OH)D, VDBP, and bioavailable 25(OH)D) to bone mineral density and lean body mass in women indoor sport athletes was examined in the current study. A strength of this study is the inclusion of high-level women indoor athletes and the analysis of VDBP and bioavailable 25(OH)D status. However, the small sample size (*n* = 36) and cross-sectional design with one time point of data collection make it difficult for statistical analyses, particularly regression analyses, to obtain statistical significance. 

## 5. Conclusions

Results demonstrated vitamin D insufficiencies across elite women collegiate indoor sport athletes. The majority of women athletes, regardless of sport, had inadequate or insufficient vitamin D status. These results may be useful in guiding nutrition professionals, coaches, and practitioners when evaluating the health status of athletes from sports with minimal exposure to sunlight. Vitamin D plays an important role in maintaining athlete health; therefore, assessments should be conducted to assess 25(OH)D levels, and when inadequacy is established, supplementation should be suggested as a vehicle to improve status. No differences were observed across teams with the exception of body composition measures, which may be attributed to the physiological demands associated with each sport. Interestingly, no relationships were found between vitamin D status and bone health. It is recommended that the relationship among 25(OH)D, VDBP, bioavailable 25(OH)D, bone mineral density, and lean body mass be investigated further in high level women athletes. 

## Figures and Tables

**Table 1:** Characteristics of participants.

Characteristic	Overall (*n* = 36)	WVB (*n* = 12)	WBB (*n* = 12)	WTF (*n* = 12)	*p*-Value
Age (years)	19.4 ± 1.4	19.2 ± 1.3	20.2 ± 1.6	19.0 ± 0.8	0.274
Skin pigmentation					0.242
Very fair/fair (41–55°)	17(47)	8(67)	6(50)	3(25)	
Medium (28–41°)	2(6)	1(8)	0(0)	1(8)	
Olive/dark (0–28°)	17(47)	3(25)	6(50)	8(67)	
Sun exposure, reported (min/day)					0.051
≤10 min	18(50)	8(66)	8(66)	2(16)	
30–40 min	14(38)	4(33)	4(33)	6(50)	
1–2 h	2(5)	0(0)	0(0)	2(16)	
≥2 h	2(5)	0(0)	0(0)	2(16)	
Dietary intake and supplementation (IU/d)	501.9 ± 417.4	342.6 ± 257.8	760.9 ± 484.2	402.3 ± 376.4	0.005
Anthropometrics					
Body height (cm)	172.75 ± 8.21	173.25 ± 5.77	177.72 ± 9.81	167.33 ± 5.21	0.005
Body mass(kg)	70.91 ± 13.22	71.81 ± 9.62	80.33 ± 15.82	60.75 ± 4.51	<0.001
Body mass index (kg/m^2^)	23.7 ± 3.2	24.1 ± 2.5	25.3 ± 4.2	21.3 ± 1.4	0.001
Body Composition					
Body fat (%)	22.9 ± 4.1	24.0 ± 3.1	25.0 ± 3.9	19.6 ± 2.7	0.001
Fat mass (kg)	17.1 ± 6.0	17.8 ± 3.9	20.9 ± 7.3	12.2 ± 2.2	0.001
Lean body mass (kg)	52.3 ± 7.3	53.2 ± 6.3	57.7 ± 7.7	47.4 ± 3.4	0.078
Whole body BMD (g/m^2^)	1.12 ± 0.20	1.12 ± 0.08	1.13 ± 0.68	1.11 ± 0.35	0.744
Whole body Z-Score	1.31 ± 0.87	1.78 ± 1.17	1.38 ± 0.33	0.80 ± 0.407	0.070
Hip BMD (g/m^2^)	1.19 ± 0.10	1.21 ± 0.13	1.21 ± 0.11	1.16 ± 0.77	0.495
Hip Z-score	1.59 ± 0.98	2.04 ± 1.29	1.71 ± 0.702	1.06 ± 0.66	0.044
Spine BMD (g/m^2^)	1.18 ± 0.11	1.18 ± 0.13	1.23 ± 0.12	1.14 ± 0.08	0.171
Spine Z-score	1.19 ± 0.99	1.31 ± 1.15	1.55 ± 0.94	0.708 ± 0.730	0.101
Biochemistry					
25(OH)D (nmol/L)	70.5 ± 32.25	87.5 ± 44.0	60.0 ± 44.0	69.5 ± 15.75	0.197
25(OH)D category					0.159
≤50 nmol/L	10(28)	3(25)	6(50)	1(8)	
50–75 nmol/L	12(33)	3(25)	4(33)	5(42)	
≥75 nmol/L	14(39)	6(50)	2(17)	6(50)	
Vitamin D binding protein (μg/mL)	437.1 ± 112.6	416.7 ± 107.5	447.8 ± 115.5	447.5 ± 122.2	0.754
Bioavailable 25(OH)D (nmol/L)^1^	4.25 ± 1.75	5.24 ± 2.0	3.25 ± 1.5	4.5 ± 1.25	0.012

Data presented as mean ± SD for continuous variables and *n* (%) for categorical. WVB, women’s volleyball; WBB, women’s basketball; WTF, women’s track and field. BMD, bone mineral density ^1^ Calculated according to previously published methods [2,6,7].

**Table 2:** Parameter estimates (β 95% CI) of association of vitamin D parameters with independent body composition variables (whole body, hip, and spine BMD, and LBM) after adjusting for sport and skin pigmentation.

Body Composition Variables	Circulating 25(OH)D (nmol/L) β (95% CI)	*p*-Value	VDBP (μg/mL) β (95% CI)	*p*-Value	Bioavailable 25(OH)D (nmol/L) β (95% CI)	*p*-Value
Whole-body BMD (g/m^2^)	0.069 (−0.216–0.354)	0.624	0.000 (−0.180–0.104)	0.703	−0.038 (−0.180–0.104)	0.586
Hip BMD (g/m^2^)	0.159 (−0.094–0.412)	0.209	0.000 (−0.001–0.000)	0.135	−0.076 (−0.205–0.053)	0.240
Spine BMD (g/m^2^)	−0.037 (−0.215–0.288)	0.766	0.000 (−0.001–0.001)	0.920	−0.067 (−0.153–0.102)	0.685
LBM (kg)	0.036 (−1.023–1.095)	0.945	−0.001 (−0.004–0.002)	0.540	−0.133 (−0.192–0.058)	0.283

**β**, unstandardized coefficient and (95% CIs). 25(OH)D, 25-hydroxyvitamin D; VDBP, vitamin D binding protein; BMD, bone mineral density; LBM, lean body mass.
